# Natural selection for earlier male arrival to breeding grounds through direct and indirect effects in a migratory songbird

**DOI:** 10.1002/ece3.1423

**Published:** 2015-02-22

**Authors:** William Velmala, Samuli Helle, Markus P Ahola, Marcel Klaassen, Esa Lehikoinen, Kalle Rainio, Päivi M Sirkiä, Toni Laaksonen

**Affiliations:** 1 Section of Ecology, Department of Biology, University of TurkuTurku, FI-20014, Finland; 2 Finnish Museum of Natural History, University of HelsinkiP.O. Box 17, FI-00014, Helsinki, Finland; 3 Natural Resources Institute FinlandItäinen Pitkäkatu 3, FI-20520, Turku, Finland; 4 Department of Animal Ecology, Netherlands Institute of EcologyDroevendaalsesteeg 10, 6708 PB, Wageningen, The Netherlands; 5 Centre for Integrative Ecology, School of Life and Environmental Sciences, Deakin UniversityWaurn Ponds, Vic., 3216, Australia

**Keywords:** Fitness, life history, microevolution, seasonal interactions, sexual selection, timing of migration

## Abstract

For migratory birds, the earlier arrival of males to breeding grounds is often expected to have fitness benefits. However, the selection differential on male arrival time has rarely been decomposed into the direct effect of male arrival and potential indirect effects through female traits. We measured the directional selection differential on male arrival time in the pied flycatcher (*Ficedula hypoleuca*) using data from 6 years and annual number of fledglings as the fitness proxy. Using structural equation modeling, we were able to take into account the temporal structure of the breeding cycle and the hierarchy between the examined traits. We found directional selection differentials for earlier male arrival date and earlier female laying date, as well as strong selection differential for larger clutch size. These selection differentials were due to direct selection only as indirect selection for these traits was nonsignificant. When decomposing the direct selection for earlier male arrival into direct and indirect effects, we discovered that it was almost exclusively due to the direct effect of male arrival date on fitness and not due to its indirect effects via female traits. In other words, we showed for the first time that there is a direct effect of male arrival date on fitness while accounting for those effects that are mediated by effects of the social partner. Our study thus indicates that natural selection directly favored earlier male arrival in this flycatcher population.

## Introduction

The annual cycle of migratory animals in temperate and arctic zones consists of at least four characteristic periods: breeding, overwintering, and two migratory periods between the respective breeding and overwintering grounds. Migrants are faced with the increasing challenge of timing each of these periods optimally in the face of ongoing changes in environmental conditions (Møller et al. 2008b; Carey 2009; Knudsen et al. 2011; McNamara et al. 2011). To understand how timing may evolve in response to these changes, knowledge about the way how selection works on the timing of these phases in the annual cycle is needed (e.g., Gunnarsson et al. 2006; Gordo et al. 2013).

In territorial migratory birds, males usually arrive to the breeding grounds earlier than females (Morbey and Ydenberg 2001), which helps them to claim and establish high-quality territories (e.g., Aebischer et al. 1996; Hasselquist 1998; Smith and Moore 2005). Early-arriving males have also been found to attract higher-quality mates (e.g., Alatalo et al. 1984; Rubolini et al. 2004), establish larger harems (Hasselquist 1998), and have more extra-pair mating opportunities (Reudink et al. 2009; Cooper et al. 2011), thereby increasing their breeding success. In addition, early-arriving males and females have been shown to perform better in breeding in terms of larger clutch sizes, more fledglings (Potti 1998; Hötker 2002; Tryjanowski et al. 2004; Sergio et al. 2007), and more recruiting offspring (Møller 1994; Hasselquist 1998).

The overall advantages of arriving early, therefore, seem to be well established for both sexes, although it has been shown that arrival date in the two sexes may also be under divergent selection (Møller 2007). However, we have generally a very limited knowledge on what the specific selection pathways are for early male arrival. Few formal analyses using directional selection differentials have been conducted on how natural selection acts on arrival time (Møller 2007; Møller et al. 2008a; Teplitsky et al., 2011; Gienapp and Bregnballe 2012; Arnaud et al. 2013). Only one study has previously considered the potential different pathways of selection on arrival date, that is, distinguishing between its direct effects on fitness and its indirect effects through earlier laying date and larger clutch size that are likely to depend on the quality of the female (Norris et al. 2004). Analyzing data from American redstarts (*Setophaga ruticilla*), Norris et al. (2004) found that the arrival date did not have a significant direct effect on the number of fledglings in either sex. Instead, the indirect fitness effects of arrival date, via female laying date and fledging date, were found to be significant. However, the path analysis of Norris et al. (2004) was not used to estimate the directional selection differential and its components that are relevant measures when comparing the strength of selection between species and traits (Scheiner et al. 2000).

To address these issues, we studied whether there is selection on the timing of male arrival in the pied flycatcher (*Ficedula hypoleuca*), a small migrant songbird breeding in Eurasia and wintering in sub-Saharan Africa. We quantify the selection on male arrival date by calculating directional selection differential using structural equation modeling (SEM), which enables us to examine to what extent selection arises through different pathways (Scheiner et al. 2000). The major benefits of applying SEM in selection studies are that it enables (1) the modeling of a more biologically realistic scenario of multivariate natural selection compared to univariate approaches and multiple regression models (Lande and Arnold 1983; Morrissey 2014) and (2) the separation of selection differentials into direct and indirect selection and their components (Scheiner et al. 2000). In other words, SEM takes into account the hierarchical sequence of the different variables, such as the causally interrelated life history events during a breeding cycle. This method is still surprisingly rarely used in studies quantifying natural selection (Morrissey 2014), despite its obvious benefits when analyzing multivariate natural selection from phenomena that are temporally structured. To our knowledge, there are no previous studies that have used this approach to model the selection on timing of migration in a migrant bird.

## Materials and Methods

### Study species

The pied flycatcher is a long-distance migrant, breeding in temperate and boreal forests from West Europe to western Siberia, and wintering in western sub-Saharan Africa. It is a small (weighting 16 g on average) insectivorous, cavity-breeding passerine bird, which spends eight months a year away from the breeding areas, either on migration or in wintering grounds in Africa. In Finland, pied flycatchers arrive between late April and early June and the last birds depart by late August. The pied flycatcher is an extensively studied model species, as it is abundant throughout its range and readily accepts man-made nest boxes, even preferring them to natural cavities. (e.g., Lundberg and Alatalo 1992)

### Study site

Our study site is situated on Ruissalo, an island of 9 km^2^ in the vicinity of the city of Turku (60°26′N, 22°10′E) in southwestern Finland. The study site consists of coniferous forest, mainly scots pine (*Pinus sylvestris*), and deciduous forest, with oak (*Quercus robur*), silver birch (*Betula pendula*), and small-leaved lime (*Tilia cordata*) as the main tree species. The study was conducted in 2005–2006 and 2008–2011. Each year, 230 timber nest boxes (inside dimensions 12.5 × 12.5 cm, inside height 23.6 cm, entrance hole 32 mm) were available and monitored, except for 2010–2011 when only 195 nest boxes were monitored. The nest boxes were set as lines along paths and roads, the nearest-neighbor nest-box distance being 20–40 meters. Each year, by the time the pied flycatchers arrived, a proportion of the nest boxes was already occupied by either great tits (*Parus major*) or blue tits (*Cyanistes caeruleus*). The number of breeding pairs of pied flycatchers in the whole study area varied annually, from 79 to 124, with a mean of 101 breeding pairs, although much fewer were available for the analysis (see below). The rest of the nest boxes were inhabited by tits or remained uninhabited. Pied flycatcher nest-box occupancy of all nest boxes thus varied from 40.5 to 53.9%, with a mean of 45.9%.

### Field observations

The arrival dates of individual pied flycatcher males to the study area were assessed by daily monitoring of the whole study area. The monitoring covered the entire period of the males’ arrival, from late April until late May. Nest-site monitoring was conducted between 7 am and 1 pm (UTC +2) by slowly walking through the study area and stopping at nest boxes, spending 3–4 min in the vicinity of each nest box. There were 2–4 observers involved in the monitoring each day, and each observer was randomly selected for monitoring a specific subset of nest boxes on a daily basis.

Flycatchers were detected using visual and auditory cues. The males can be individually and unambiguously recognized based on the presence of colored and aluminum rings, and plumage details, including coloration of the back, rump pattern, shape and size of the white forehead patch, and the amount of white on the tail and wings. The shape of the forehead patch was characterized as a uniform block, separate dots, or linked dots. The size of the forehead patch was assigned as large, medium, small, or very small/absent. Dorsal coloration was assessed using a scale from 1 to 7 designed by Drost (1936): 1 being black and 7 being female-like brown. In addition to character determination using binoculars, digital photography with a telephoto lens was used to record individual plumage details in 2010 and 2011.

The arrival date estimate is the first date a male was observed in the study area during the daily monitoring. We included only those individuals that stayed breeding in the study area and whose identity could later be confirmed on plumage characters while caught at the nest box they were breeding in (see reasoning below). To avoid influencing the males’ territory selection, they were not trapped and ringed or otherwise interfered with during this period (except in 2009 when males were captured some days after arrival). Instead, each male was studied with binoculars for individual identification. Females were not included in the study because, due to the extremely fine differences in plumage characters, they cannot be reliably identified individually, making the determination of exact arrival day impossible without interference (i.e., catching and ringing).

The breeding performance of each male and his mate was monitored throughout the breeding season using the following indicators: laying date, clutch size, and number of fledged young (Table1). Both adults were captured when the chicks were 6–7 days old. While in the hand, the identity of a male was confirmed to relate to the same territorial male that was observed in the vicinity of the nest box during the arrival period. From the whole breeding population, we excluded the ones who abandoned their nest or whose nests were predated at an early stage (and thus, their identity could not be verified), or who were randomly chosen to other study experiments that might compromise mate choice, clutch size, or number of fledglings.

**Table 1:** Descriptive statistics of the variables used in the structural equation modeling, pooled over the whole study period. In the selection analysis, however, annual trait means were used in standardization. In male arrival date and female laying date, the first event was given value the 0, and subsequent events are days after the first event

	Mean	Min–max	SD	*N*	% missing values
Arrival date	13.40	0–31	5.74	363	–
Laying date	14.70	0–30	4.42	363	–
Clutch size	6.56	2–9	0.80	363	–
Number of fledglings	5.70	0–9	1.50	261	28.1

### Statistical analyses

**Figure 1:**
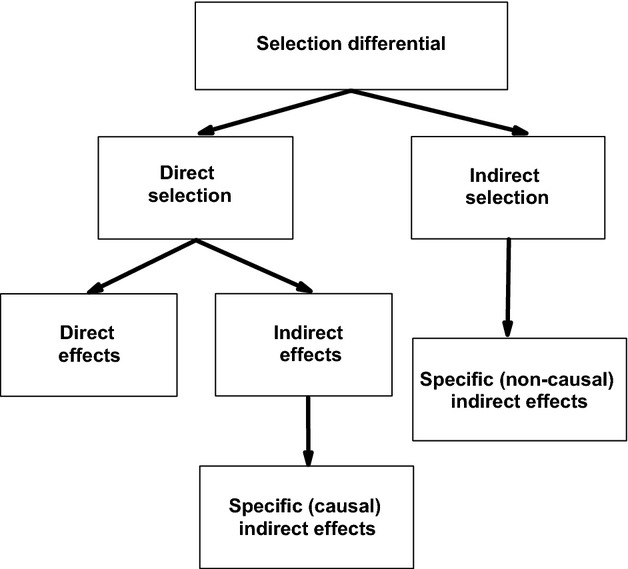
The partitioning of selection differential into direct and (noncausal) indirect selection. Direct selection can be further decomposed to direct effects (i.e., selection gradients) and to indirect effects. Depending on a model structure and complexity, indirect effects of direct selection as well as indirect selection can be further decomposed to their respective specific indirect effects.

We used structural equation modeling (SEM), or in this case path analysis, to study the strength of natural selection on male arrival date, female laying date, and clutch size by estimating directional selection differentials for these traits (Scheiner et al. 2000). The logic of using SEM in studies of natural selection is shown in Figure1. The relative number of offspring produced (fledglings) was used as annual fitness measure here. The directional selection differential is estimated by the covariance between a trait and fitness (Fig.1). It can be decomposed into direct and indirect selection, which, in turn, may involve several different pathways, depending on model complexity (Scheiner et al. 2000). In SEM framework, direct selection on a trait is estimated by summing its direct effect on fitness and indirect effects through traits that go forward toward fitness in a SEM diagram (Scheiner et al. 2000; Fig.1). If several such mediating traits are included in the model, the indirect effect of direct selection can be further decomposed into specific indirect effects (Fig.1). The direct effect of a trait on fitness is the selection gradient, or the slope of the (partial) regression coefficient (Lande and Arnold 1983). Indirect selection (or noncausal selection due to shared causes) on a trait is estimated by summing its effects through backward-going traits that are connected to fitness in a path diagram (Scheiner et al. 2000). Again, depending on model complexity and on the position of a variable of interest in a structural model, indirect selection (like direct selection) can be decomposed into specific indirect effects (Fig.1).

**Figure 2:**
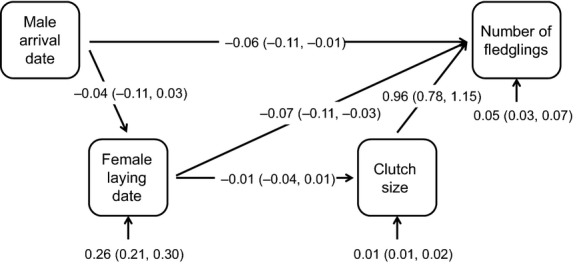
The structural equation model used to estimate directional selection differential on male arrival date, female laying date, and clutch size. Relative number of fledglings was used as a proxy for fitness. Arrows between boxes represent assumed causal associations between the mean-standardized traits, and open short arrows denote residual variances of dependent variables. Direct effects (paths) from a trait to fitness can be considered selection gradients. Numbers in parentheses are 95% confidence intervals.

Based on the ecology of migrant birds and on the questions asked in this study, we formulated an *a priori* structural equation model assuming that the male arrival date has a direct effect on fitness as well as an indirect effect on fitness through both female laying date and clutch size (Fig.2). The model also assumes that the female laying date has a direct effect on fitness but also an indirect effect via clutch size and that clutch size has a direct effect on fitness only (Fig.2). Because the traits included in our analysis differed in their measurement scale and sample variance (Table1), we followed Hereford et al. (2004) to obtain mean-standardized selection estimates and divided all traits (including fledgling number to measure relative fitness) by their annual trait means prior to analysis. The resulting mean-standardized selection coefficients represent the proportional change in fitness for a proportional change in the mean of the trait in question. This makes it possible to compare our results with other traits, populations, or species more reliably had we used standardization based on phenotypic standard deviations (Hereford et al. 2004; Matsumura et al. 2012). However, mean standardization requires variables with natural origin that can be established as equal within different studies (Hereford et al. 2004). Therefore, for both male arrival date and female laying date, the date of the first event (i.e., the arrival date of the first male or the first laying date of the season) was given the value of zero and the subsequent events were scored by days since that event on a yearly basis (Houle et al. 2011). However, we use terms “arrival date” and “laying date” (instead of “day”) throughout the article in spite of the above-mentioned transformation.

The fit of the *a priori* model to the observed data was examined using the chi-square test (*χ*^2^) and the following fit indices: the root mean square error of approximation (RMSEA), standardized root mean square residual (SRMR), the comparative fit index (CFI), and the Tucker–Lewis index (TLI) (West et al. 2012). Both RMSEA and SRMR are badness-of-fit measures, where 0 indicates a perfect fit for the model. In contrast, in both CFI and TLI, a value approaching 1 indicates good model fit (West et al. 2012). RMSEA has the added benefit of providing 90% confidence intervals for the estimate, and it can be used to test the null hypothesis that the estimate is <0.5, indicating a good fit (West et al. 2012). The rough cutoff values used to indicate a well-fitting model for SRMR, CFI, and TLI were <0.08, >0.95, and >0.95, respectively (West et al. 2012). As we had repeated observations for some males (*n *=* *42) from different years, we used a design-based clustering method that corrects for nonindependence of data points by adjusting parameter SEs without explicitly estimating this dependency, as carried out in mixed modeling (Kalton 1977). Model parameters were estimated using robust maximum-likelihood estimation (MLR) that allows for non-normal response distributions (here, the relative fledgling number had a skewness of −1.41 and a kurtosis of 2.47), and missing data were handled using full-information maximum likelihood (FIML). Analyses were conducted with Mplus (version 7.3; Muthén and Muthén 1998–2012).

## Results

The *a priori* structural equation model showed good fit to the data (*n *=* *363, *χ*^2^_mlr _= 0.24, *df *= 1, *P *=* *0.63; RMSEA (90% CIs) = 0.00 (0.00, 0.11), *P *=* *0.75; CFI = 1.00; TLI = 1.08; SRMR = 0.007). The model indicated statistically significant directional selection differential for earlier male arrival date and female laying date and for larger female clutch size (Table2). A 10% shift toward an earlier male arrival date and female laying date gives an expected proportional increase in fitness by 0.56% and 0.81%, respectively (Table2). A 10% larger clutch size increases fitness by 9.65% (Table2). The directional selection differentials for male arrival date and female laying date did not statistically differ from each other (*z *=* *0.68, *P *=* *0.50), but selection differential for clutch size was significantly stronger than that of male arrival date (*z *= −10.7, *P *<* *0.0001) and female laying date (*z *= −10.6, *P *<* *0.0001).

**Table 2:** The proportional model-predicted directional selection differential for male arrival date, female laying date, and clutch size on number of fledglings (i.e., fitness), decomposed into direct and indirect selection, where direct selection is further decomposed into direct and indirect effects. Numbers in parentheses are 95% confidence intervals

	Arrival date	Laying date	Clutch size
Directional selection differential	−0.056 (−0.108, −0.005)	−0.081 (−0.129, −0.034)	0.965 (0.783, 1.147)
Direct selection	−0.056 (−0.108, −0.005)	−0.084 (−0.132, −0.036)	0.964 (0.782, 1.146)
Direct effect (or selection gradient)	−0.059 (−0.111, −0.008)	−0.070 (−0.114, −0.027)	0.964 (0.782, 1.146)
Indirect effects	0.003 (−0.003, 0.009)	−0.013 (−0.034, 0.007)	–
Indirect selection	–	0.002 (−0.003, 0.007)	0.001 (−0.001, 0.002)

**Figure 3:**
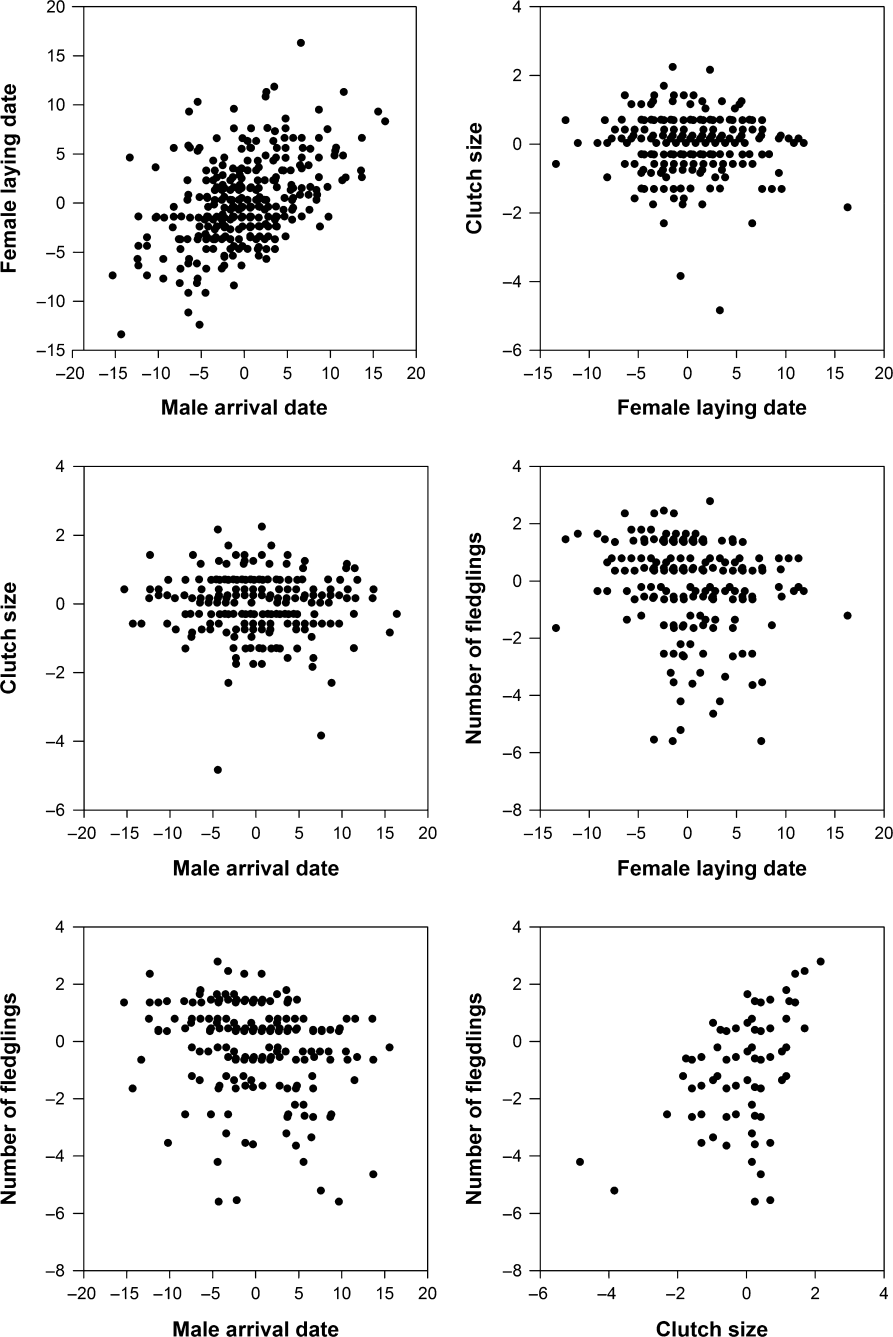
Associations between all traits used in the structural equation modeling (SEM). All values are standardized by their annual mean.

Indirect selection for male arrival date was not defined in our model, and thus, its directional selection differential equaled direct selection (Table2). The estimates of indirect selection for female laying date and clutch size were very small and statistically nonsignificant, showing that directional selection differential for these traits was mainly due to direct selection (Table2). Selection gradients (i.e., direct effects) for all traits were statistically significant (Fig.2; Table2). The total indirect effects of direct selection through forward mediating traits included in the model were not statistically significant for any of the traits studied (Table2), indicating that, for example, selection for male arrival date was owing to its direct effect on fitness. The pairwise associations in the raw data between all traits used in the analysis (male arrival date, female laying date, clutch size, and number of fledglings) are shown in Figure3.

## Discussion

We investigated the strength of natural selection on timing of male spring arrival in the pied flycatcher by taking advantage of structural equation modeling (SEM). SEM enabled the realistic modeling of selection episodes on temporally ordered breeding traits. We found directional selection differential for an earlier male arrival date. Importantly, this selection coefficient resulted from the direct effect of male arrival date on fitness and not from the indirect effects on fitness through female traits, that is, laying date and clutch size. The magnitude of selection on the male arrival date was similar to selection for earlier female laying date. Both of these were, however, several orders of magnitude lower than selection for larger clutch sizes, which logically arises from the strong correlation between clutch size and number of fledglings in the current sample.

A wealth of previous studies has shown correlations between the timing of breeding (i.e., laying date) and breeding success (for the pied flycatcher, see, e.g., Lundberg and Alatalo 1992; Canal et al. 2012), including the associations between male arrival date and reproductive success (e.g., Alatalo et al. 1984; Aebischer et al. 1996; Hasselquist 1998; Norris et al. 2004; Rockwell et al. 2012). However, only few studies have investigated how natural selection acts on spring arrival dates by quantifying selection coefficients (Møller et al. 2008a; Gienapp and Bregnballe 2012; Arnaud et al. 2013), and even fewer have examined the pathways linking arrival time with fitness. Norris et al. (2004) studied the American redstart and estimated the different pathways of selection on arrival date to fitness due to direct and indirect effects, but they did not examine the strength of natural selection (i.e., the directional selection differential) for male arrival date or for female traits. The current study therefore seems to be the first one fully utilizing the insight obtained from the SEM for studying natural selection on timing of spring arrival in a migrant bird.

Our results show, for the first time, a direct effect of male arrival date on fitness while accounting for those indirect effects that are mediated by potential effects of the social partner. In other words, directional selection differential and direct selection for earlier male arrival were due to its direct effect on fitness and not due to its indirect effects via female traits. While female traits most likely influence breeding performance, for example, through timing of laying, clutch size, and level of parental care, the male arrival date seems to have a direct effect on fitness. Our results thus suggest that early-arriving males are either of a higher phenotypic quality, have better genes, or are able to gain better resources, such as high-quality breeding territory, compared to later-arriving males.

A review by Hereford et al. (2004) used 38 studies that had reported the necessary information for the postcalculation of mean-standardized selection gradients (of which nine were on birds). In those 38 studies, the median absolute value of multivariate mean-standardized selection gradients was 0.86 for life history traits, 0.38 for fecundity, and 0.54 for all estimates (Hereford et al. 2004). The selection gradients (the direct effects) for the male arrival date and female laying date (−0.06 and −0.07, respectively) in our study can thus be considered minor. In contrast, the selection gradient for clutch size (0.96) indicates strong direct selection. However, comparing selection gradients, or selection differentials in particular (Scheiner et al. 2000), in our study to those presented in Hereford et al. (2004) is not straightforward due to methodological differences. In the current study, we have taken into account the temporal structure of the pied flycatcher breeding cycle, which gives a biologically more realistic and causally defined model of natural selection, whereas multiple regression models, assuming no such hierarchy between traits, are traditionally used in selection analyses (Hereford et al. 2004; Morrissey 2014). Although not fully excluding the possibility of equivalent models (i.e., models assuming a different causal structure that also show similar fit to the data), our model fitted the data well, suggesting that the selection estimates obtained can be considered to have low bias. We therefore encourage future studies of natural selection to use SEM in order to base the selection estimates on more causally plausible models of natural selection in the wild.

It should be noted that our study only considers selection on parental fecundity but not on survival. There may also be costs of, and selection against, early migration and arrival due to harsh weather and food shortage (Møller 1994; Kokko 1999; Brown and Brown 2000; Møller et al. 2008b). Unfortunately, these are extremely difficult questions to study in a small songbird, such as the pied flycatcher, until it is possible to track them individually throughout their annual cycle. The importance of the trade-offs between the potential survival risks associated with early migration and the benefits of early arrival to breeding grounds thus remain to be studied. It also remains to be examined how individual condition (state) affects these trade-offs: The risk may be more modest if early-arriving males are also higher-quality individuals in the sense that they are more likely to outlive disadvantageous conditions (e.g., Møller 1994). In fact, the advantage of early breeding is emphasized when temperature prior to breeding is low, possibly indicating higher-quality individuals to be less influenced by harsh conditions (Ahola et al. 2012).

It can be argued that the number of recruits could have been used as a fitness proxy instead of the number of fledglings, but several authors have concluded that fledgling number is a relevant fitness measure (e.g., Brommer et al. 2004) or even argued against assigning fitness across generations (e.g., Lande and Arnold 1983; Cheverud and Moore 1994; Wolf and Wade 2001). In addition, in this species, the dispersal distribution is particularly wide (Lundberg and Alatalo 1992; Lehikoinen 2014) and, consequently, the numbers of recruits recorded at parental breeding grounds are low. Therefore, the number of recruits is not an appropriate or reliable fitness measure in this species.

In conclusion, we have shown that there is directional selection for early male arrival to breeding grounds in a migratory bird. By taking into account the sequential nature of the fecundity traits in question, we estimated the strength of selection in a standardized manner as well as defined the specific pathways of directional selection for earlier male arrival date. We wanted to study whether an association between arrival date and fledgling number might arise only through female traits, such as the laying date or clutch size, but it does not seem to be so. As our study emphasizes, the timing of different periods within the annual cycle can clearly be associated with direct fitness consequences. By studying how natural selection acts on each of these periods, it will be possible to identify what the relative importance is of these periods for birds, and gain important knowledge for evaluating the potential of migrants to cope when the environment changes.
